# Cotton Yield Estimation From Aerial Imagery Using Machine Learning Approaches

**DOI:** 10.3389/fpls.2022.870181

**Published:** 2022-04-26

**Authors:** Javier Rodriguez-Sanchez, Changying Li, Andrew H. Paterson

**Affiliations:** ^1^Bio-Sensing and Instrumentation Laboratory, College of Engineering, The University of Georgia, Athens, GA, United States; ^2^Phenomics and Plant Robotics Center, The University of Georgia, Athens, GA, United States; ^3^Plant Genome Mapping Laboratory, The University of Georgia, Athens, GA, United States

**Keywords:** cotton yield estimation, machine learning, UAS, SVM, remote sensing

## Abstract

Estimation of cotton yield before harvest offers many benefits to breeding programs, researchers and producers. Remote sensing enables efficient and consistent estimation of cotton yields, as opposed to traditional field measurements and surveys. The overall goal of this study was to develop a data processing pipeline to perform fast and accurate pre-harvest yield predictions of cotton breeding fields from aerial imagery using machine learning techniques. By using only a single plot image extracted from an orthomosaic map, a Support Vector Machine (SVM) classifier with four selected features was trained to identify the cotton pixels present in each plot image. The SVM classifier achieved an accuracy of 89%, a precision of 86%, a recall of 75%, and an F1-score of 80% at recognizing cotton pixels. After performing morphological image processing operations and applying a connected components algorithm, the classified cotton pixels were clustered to predict the number of cotton bolls at the plot level. Our model fitted the ground truth counts with an *R*^2^ value of 0.93, a normalized root mean squared error of 0.07, and a mean absolute percentage error of 13.7%. This study demonstrates that aerial imagery with machine learning techniques can be a reliable, efficient, and effective tool for pre-harvest cotton yield prediction.

## 1. Introduction

Cotton is a major industrial crop in the United States (U.S.), especially in the southern and western U.S. Cotton fiber is one of the principal natural textile fibers worldwide (Townsend and Sette, 2016), and the U.S. is the third leading cotton producer with an expected production of 22.5 million bales for 2019/20, just after China (27.5 million bales) and India (27 million bales). Cotton is a soft staple fiber that grows from the surface of seeds, enclosed in pods known as bolls. Primary components of economic yield, cotton boll number and boll weight are agronomic traits that help to define cotton crop performance in its last stages of growth. These traits can be used as indicators of fiber production, which ultimately play a key role in breeding programs and may also provide valuable information for farmers to plan hedging strategies.

Lint yield is one of the most important criteria for selecting new lines in breeding programs (Bourland and Myers, 2015), but it is costly to obtain reliable data. Visual estimation of yield performance is often used by cotton breeders to select promising cotton genotypes, but it can be challenging. Morphological characteristics of cotton plants such as general shape, branch density, or leaf area change during the growth cycle of the crop and they may mislead visual ratings of yield (Bourland and Myers, 2015). Moreover, boll size can vary by year, breeding line, and position on each fruiting branch, which can make it difficult to standardize visual cotton yield quantification methods. Physical harvesting of the bolls, either manually or by using mechanical pickers, to reduce the estimation bias is labor intensive and time-consuming, limiting the number of plots that can be quantified (Bowman et al., 2004). Thus, the development of tools for effectively automating plant phenotyping tasks is of great potential value for breeding programs.

In recent years, the applications of unmanned aerial systems (UAS) in agriculture have grown rapidly and have transformed modern farming. UAS are relatively inexpensive and can be equipped with a variety of sensors, which makes them a valuable tool for large crop field monitoring. These systems can be programmed easily to navigate pre-defined paths with a specific velocity while retaining a specific distance from the crop. This means that they can be used to collect data remotely from the field at optimal resolutions quickly and easily. UAS surveying has been widely used for monitoring different crops (Barbedo, 2019), but only a few studies have addressed the use of these systems to estimate cotton yield, and only two have investigated their use for cotton genotype selection. The methodologies used to estimate cotton yield from UAS imagery can be classified in two main groups: approaches based on the use of only 3-channel RGB (Red, Green, Blue) color images, and approaches that utilize a combination of different sensor data to indirectly calculate lint yield.

To estimate cotton yield from RGB imagery, one of the main techniques is color thresholding segmentation, which has been applied either to a single color channel or to multiple channels at the same time. For instance, Dodge (2019) applied a global thresholding method (using a fixed threshold value) to the B channel alone on RGB aerial images to isolate cotton-related pixels. Their methodology achieved an *R*^2^ of 0.817 for the first year's experimental data. However, this relationship was not consistent, and they needed to include additional postprocessing to improve their model generalization for the next year's data (*R*^2^ = 0.736). Yeom et al. (2018) analyzed the spatial and spectral characteristics of open cotton bolls on RGB images during the harvest period. They established a global automatic threshold based on Otsu's method to separate cotton bolls from other non-target objects. They achieved *R*^2^ values of 0.63–0.65 at estimating yield using the cotton boll area as the input variable. However, they assumed that cotton bolls have higher spectral values than the other elements of the crop, which can be a limiting factor with changing illumination conditions when the range of image intensities of the color channels for the cotton bolls can resemble other crop elements. Huang et al. (2016) found that a global RGB threshold could not extract the cotton pixels from the images accurately because the range of image intensities in the R, G, and B channels of the cotton bolls overlapped with that of the soil and other crop elements. Alternatively, they proposed the application of the thresholding technique on Laplacian images obtained from the divergence of the gradient of each image with respect to pixel intensity. They were able to establish a ratio—cotton unit coverage (CUC)—of the number of cotton boll pixels detected to the number of pixels in a particular area. They achieved their best results (*R*^2^ = 0.83) after introducing additional postprocessing steps to detect and remove poorly illuminated plot images because their method was affected by shadowing and changing illumination conditions.

Additional approaches based only on RGB images have also been proposed. Maja et al. (2016) estimated cotton yield of small field plots from a cotton breeding program using K-means clustering algorithm with 4 classes. They clustered the cotton pixels on the image based on their color and found a linear relationship (*R*^2^ = 0.782) between the ratio of cotton pixels with respect to the total image area and the actual yield. However, they needed to introduce a fixed cluster size constraint to avoid large clusters and reduce misclassification of highly reflective areas of the scene such as the bare soil. This additional constraint can limit the generalization of their methodology for highly dense cotton crops where the cotton bolls tend to form large groups. Chu et al. (2016) estimated additional crop information (plant height and canopy cover) from aerial RGB images and were able to model cotton yield (*R*^2^ = 0.529) before maturation and boll opening. However, their study of yield was limited to the stages of the crop before defoliation such that the canopy cover could be computed correctly.

In addition to RGB cameras for lint yield estimation, multispectral and thermal cameras have been used. Huang et al. (2013) used various vegetation indices obtained from multispectral aerial images in conjunction with soil properties to estimate yield variation. Their model based on the ratio of vegetation index (RVI) and soil electrical conductivity (EC) measurements performed well for non-irrigated fields (*R*^2^ = 0.718) but was unable to estimate yield accurately for irrigated fields (*R*^2^ = 0.033). Feng et al. (2020) modeled cotton yield using multiple features derived from RGB, multispectral, and thermal cameras. They applied a global threshold in all three channels R, G, and NIR (near infrared radiation) to discriminate open cotton bolls from the soil and leaves. They found that the ratio of the number of cotton pixels to the overall number of pixels in a specific area of a multispectral image (cotton fiber index, CFI) could be used to estimate yield at the pre-harvest stage (*R*^2^ = 0.90). Moreover, by using a combination of plant height, CFI, canopy temperature and the a^*^ component of the CIELAB color space, they obtained an even better result (*R*^2^ = 0.94). However, this sophisticated approach required the simultaneous use of color, multispectral and thermal cameras, which is costly and may require more computation capacity, time, and labor for data collection and processing.

Methods based on machine learning (ML) techniques have been explored recently. Support vector machines (SVM) are one of the most widely used machine learning algorithms for supervised data classification and regression analysis. Based on statistical learning, SVMs aim to identify a decision boundary to partition data in a high-dimension feature space into two sets. This decision hyperplane can be then used for data classification or regression analysis. There are two basic SVM formulations to perform these tasks. For data classification, support vector classification (SVC) models (Cortes and Vapnik, 1995) try to find the hyperplane to separate the input data belonging to two different classes with the maximum margin. The learning process for an SVC aims to maximize that margin and minimize classification errors between the two classes. These classification models return for each input data a class label and its probability of belonging to each class. The second type of SVM is for regression problems. Support vector regression (SVR) is a regression function that can predict dependent variable by using independent variables as continuous values instead of class labels. The SVR works with the similar principle as SVM: to find the hyperplane that best fits the data inside a decision boundary delimited by a predefined error margin (Drucker et al., 1996; Vapnik et al., 1997). The models can be optimized using the regularization parameter *C* and the margin of tolerance ϵ. During the last decade, SVMs and its variants have been successfully applied in agricultural remote sensing for crop classification (Song et al., 2014; Liu and Whitty, 2015) and plant disease identification (Rumpf et al., 2010; Garcia-Ruiz et al., 2013; Raza et al., 2015). For cotton crops, SVMs have been successfully used to identify cotton flowers from multispectral imagery (Xu et al., 2019) and for budding rate monitoring from aerial RGB imagery (Xia et al., 2019). Regarding cotton yield estimation, other ML methods have also been used. By using RGB and multispectral aerial imagery, Ashapure et al. (2020) implemented an artificial neural network (ANN) to estimate cotton yield throughout the season based on crop canopy attributes. They were able to predict cotton yield at early stages of the crop with an average *R*^2^ = 0.861 using features that included canopy information, multispectral vegetation indices, cotton boll information (obtained by using a color-based threshold method, Jung et al., 2018), and crop irrigation status. This advanced methodology required the use of color and multispectral imaging systems, which increases platform costs and would entail additional time and labor for data collection and processing.

Methods for cotton yield estimation based on remote sensing techniques have various limitations. Traditional image processing techniques, such as thresholding, are not flexible enough to adapt to variable conditions present in the scene, resulting in relatively low performance. Some studies rely upon additional sensors to retrieve new features, which ultimately increases platform cost and usage and processing complexity. In this article, we present a simple and easy-to-implement methodology for estimating pre-harvest cotton yield at the plot level. By using RGB airborne imagery and a SVM algorithm, this new methodology could benefit cotton breeders by allowing them to acquire accurate information about different crop plots for their selection experiments in a timely manner. Moreover, it also may provide farmers an inexpensive, quick, and precise estimation of the yield in their cotton fields before harvesting. The specific objectives of this study were to (1) implement a support vector machine model to classify cotton boll pixels in aerial images; (2) develop a predictor to estimate the number of cotton bolls at the plot level; (3) evaluate the performance of the proposed method against ground truth yield measurements; and (4) apply the proposed method to identify differences between genotypes in a cotton breeding trial.

## 2. Materials and Methods

### 2.1. Experimental Field

**Figure 1:**
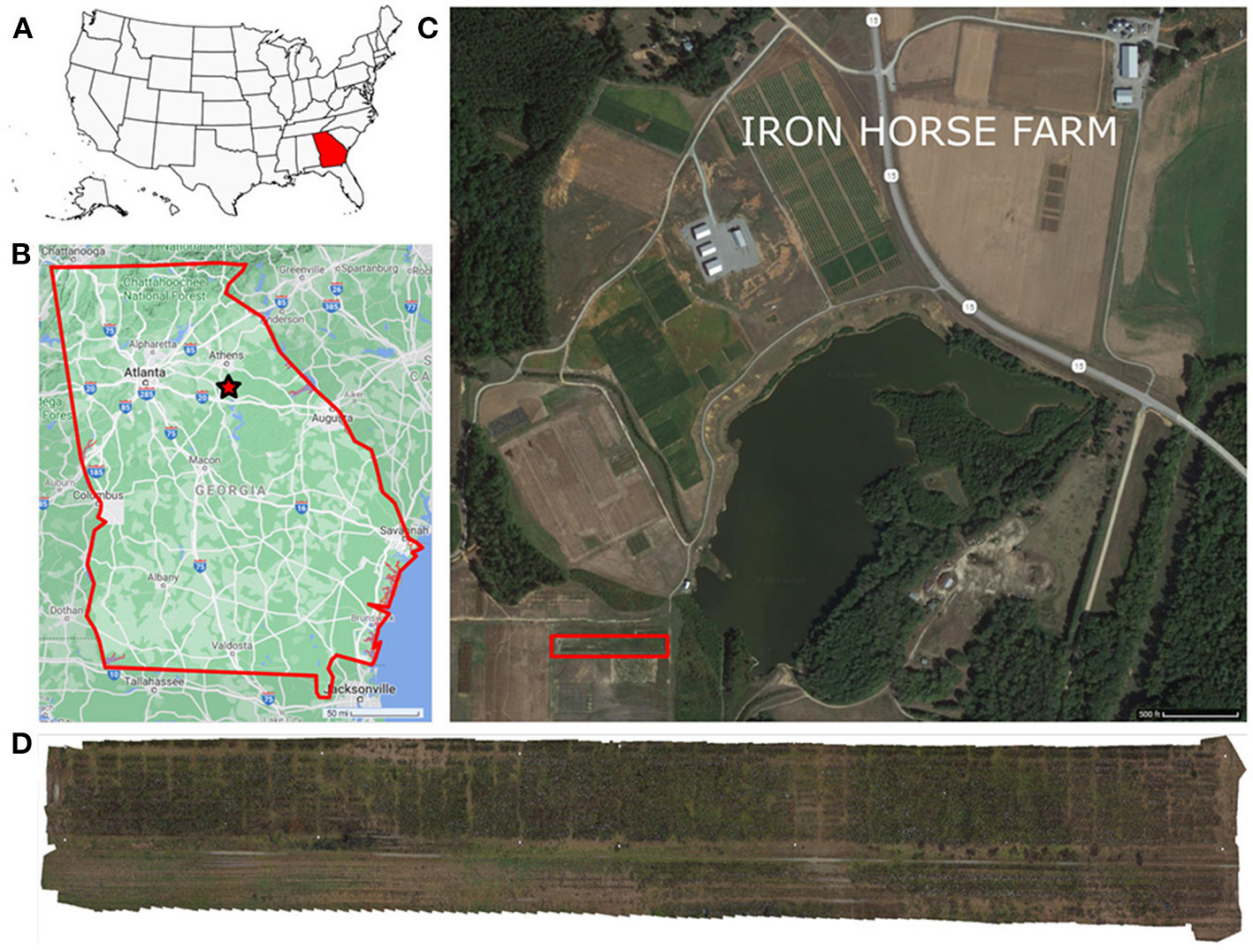
Experimental field location. The experiment was conducted in the Iron Horse Farm, Greene County, GA, U.S. **(A)** Location map of Georgia, marked in red, in the U.S., **(B)** General map of Georgia, the experiment location is marked with a red star, **(C)** Iron Horse Farm aerial view (Greene County, GA), the specific location of the experimental field is delimited by a red rectangle, **(D)** Field layout.

The field under study was located at the Iron Horse Farm (IHF) in Greene County, Georgia, U.S. (33°43′01.3^′′^N 83°18′29.1^′′^W) (Figure 1). The dimensions of the field were 200 × 12m (length × width), and it comprised a total of 488 plots. These plots were arranged into 10 rows with 46 plots per row and 1 additional row of 28 plots. A total of 220 different cotton genotypes were planted in plots of approximately 3-meters wide, with a final plant density dependent on the germination rate of the seeds. Six of the genotypes were commercial cultivars (TAM94L25, Acala1517-98, UA48, FM832, DeltaPine393, and GA230) with 10 replications per cultivar. The rest were 214 breeding lines from 10 different cotton populations (J, K, L, M, N, O, P, Q, R, S) with two replicates.

### 2.2. Data Collection

#### 2.2.1. Aerial Imagery

**Figure 2:**
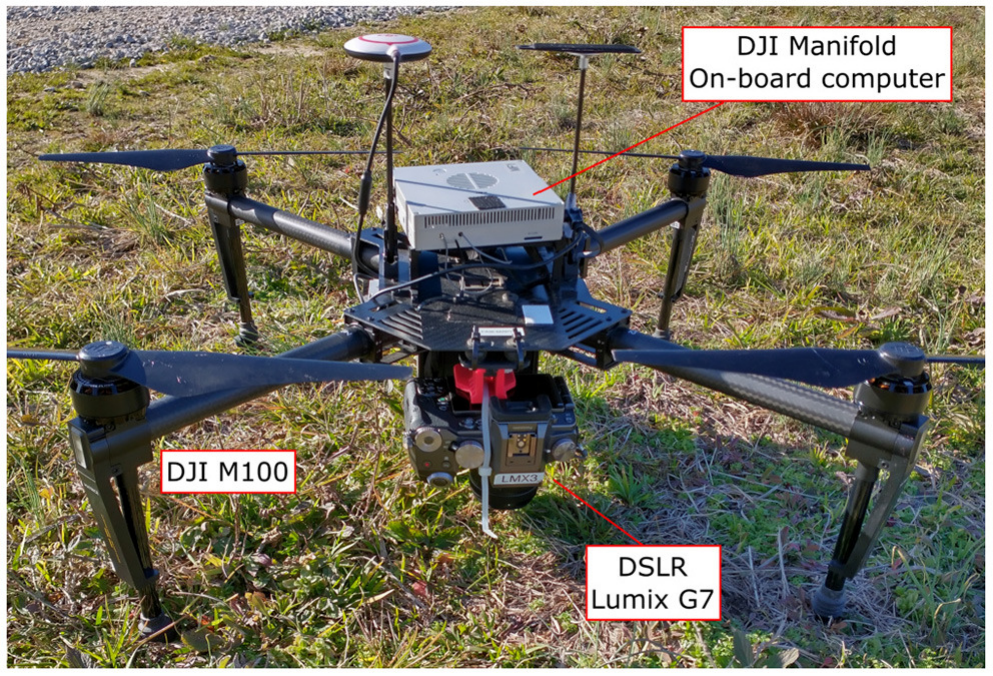
Equipment used for image collection. Unmanned Aerial Vehicle, DJI Matrice M100 Pro, equipped with a Manifold onboard computer and a Panasonic Lumix DMC-G7 DSLR camera as imaging device.

Original RGB color images were captured on February 1, 2020 on a single flight using a quadcopter DJI Matrice M100 (Shenzhen DJI Sciences and Technologies Ltd., Shenzhen, China), equipped with a Lumix G7 digital single-lens reflex (DSLR) camera (Panasonic Corporation, Osaka, Japan). This camera has a 17.3 × 13 mm CMOS image sensor with 16.0 megapixels (4592 × 3448 pixels) resolution and stores captured images using the sRGB color space. The camera was mounted on the bottom of the drone using a custom 3D-printed bracket, which ensured that the camera lens was aligned to a 90 degree angle relative to the ground. Figure 2 shows the system used for data collection. The flight was controlled internally by the M100's N1 flight controller and was carried out at a height of 15 m above ground level, and at a speed of 1.9 m/s. With this configuration, the ground pixel size was 0.26 cm/pixel. The camera was configured automatically according to the light conditions of the field. Different white balance configurations were tested for color balancing before the flight, and the “Auto White Balance” compensation (AWB) was found adequate for the weather conditions on the collection day. A Manifold onboard computer (Shenzhen DJI Sciences and Technologies Ltd., Shenzhen, China) was in charge of triggering the camera at a constant rate of 1 frame per second. The forward overlap between images in the same flight line was configured to 80%, while the side-by-side overlap between adjacent flight lines was set to 60%. A total of 447 images were collected during the flight.

**Figure 3:**
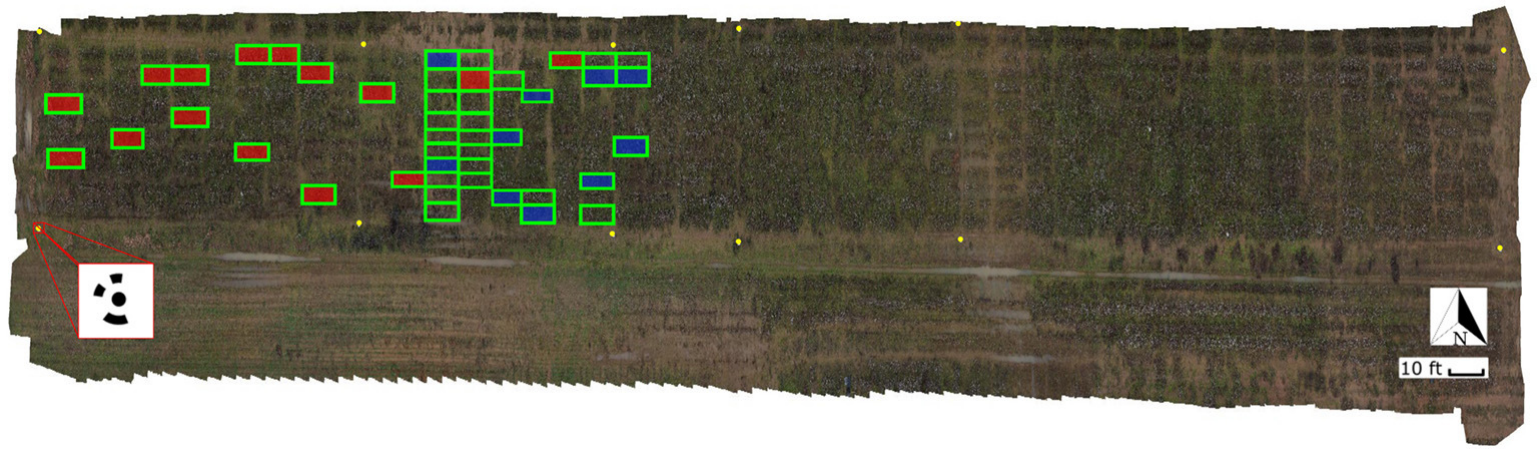
General orthomosaic map of the experimental field. Ground Control Points (GCP) used for image geo-referencing are identified as yellow circles; a total of 12 GCPs were used around the field. The red square at the bottom-left corner shows a zoom-in view of one of the 60 × 60 cm square GCPs with the 12-bit circular coded pattern. Ground truth plots measured on February 2, 2020 using digital approaches are identified with green boxes (45 plots). Plots harvested on February 3, 2020 are identified with boxes filled in red (15 plots); ground truth plots harvested on February 27, 2020 are identified with boxes filled in blue (10 plots).

A set of 12 ground control points (GCP) with circular patterns were generated using Agisoft Metashape software (Metashape Professional 1.5.5, Agisoft LLC, Russia) and deployed along the field's border for geo-referencing the UAS images (Figure 3). The patterns were cut off from matte black adhesive-backed vinyl sheet and pasted onto a white acrylic 60 cm square sheet. The GCPs were surveyed in the field using an RTK-GPS system.

#### 2.2.2. Ground Truth Data

A digital method was used to provide the ground truth data to evaluate the remote sensing predictions. Since manual harvesting of cotton bolls is time consuming, labor intensive, and destructive, we employed high resolution 3D point clouds and a virtual reality (VR) annotation tool to count number of bolls digitally. The field was scanned on February 2^*nd*^ using terrestrial laser scanning (TLS) techniques. A FARO Focus S70 3D laser scanner (FARO Technologies Inc., Florida, U.S.) was used to collect high resolution 3D point cloud data (PCD) from multiple locations through the field. The scanner was configured to $\frac{1}{2}$ resolution, and 2 × quality. With these parameters a full scan can collect up to 174.8 megapoints with a point distance of 3.1 mm in a scan distance of 10 meters. The LiDAR data was captured from the ground at a distance ranging between 1 and 2 m from the plants, to enable the visual identification and counting of the cotton bolls. Individual scans were registered as a single PCD using FARO SCENE 2019 software (FARO Technologies Inc., Florida, U.S.). A set of 10 coded planar markers obtained from SCENE software were deployed around the field to facilitate the coregistration of multiple PCDs. SCENE was configured to automatically detect the registration markers and align the scans. After registration, individual plots were manually segmented and extracted from the 3D reconstructed PCD as .PTS files using SCENE *clipping box* tool. Each individual plot was then processed using CloudCompare software (version 2.11.2). A statistical outlier removal using 2 points for mean distance estimation and 1.0 as the standard deviation multiplier threshold was applied to the individual point clouds to reduce noise and clean spurious points. From the 488 plots that composed the field, a set of 45 plots (Figure 3) were selected for digital ground truth counting. A VR annotation tool developed by the Virtual Experience Laboratory at the University of Georgia was leveraged to count the cotton bolls from the clean PCDs using an Oculus Quest 2 VR set (Facebook Technologies Inc., California, U.S.) and a desktop computer. More details of this VR tool will be covered in a future manuscript. These counts were considered as the ground truth measurements (GT_VR_) for further analysis.

The plots used as the ground truth included a representative subset of the plots in the field: from plots with a small number of cotton bolls (<20 cotton bolls) to highly dense cotton plots (>350 cotton bolls). A summary of the ground truth values for both the manual and digital sampling is presented in Table 1. To calibrate this approach, the digital ground truth counts were regressed against the actual number of cotton bolls in a subset of manually harvested plots. The manual ground truth subset was composed of 25 plots that were randomly selected from the 45 plots in the digital ground truth set. In these plots, only the open cotton bolls were harvested, counted, and weighted manually. These manual ground truth measurements (GT_manual_) were performed in two different batches: 15 plots were harvested on February 3^*rd*^, 2020 and 10 plots were harvested on February 27^*th*^, 2020 (Figure 3). A strong linear relationship (*R*^2^ = 0.996) was found between GT_VR_ and GT_manual_ for the 25 manually harvested plots.

**Table 1:** Ground truth data summary.

	**Samples no**.	**Min (boll number)**	**Max (boll number)**	**Mean (boll number)**
Digital ground truth	45	14	362	160.933
Manual ground truth	25	61	367	184.680

*Manual ground truth counts include the cotton boll number measured by destructive sampling of 25 plots randomly selected from the field. Digital ground truth counts include the cotton boll number of 45 ground truth plots measured using digital approaches*.

### 2.3. Data Processing Pipeline

**Figure 4:**
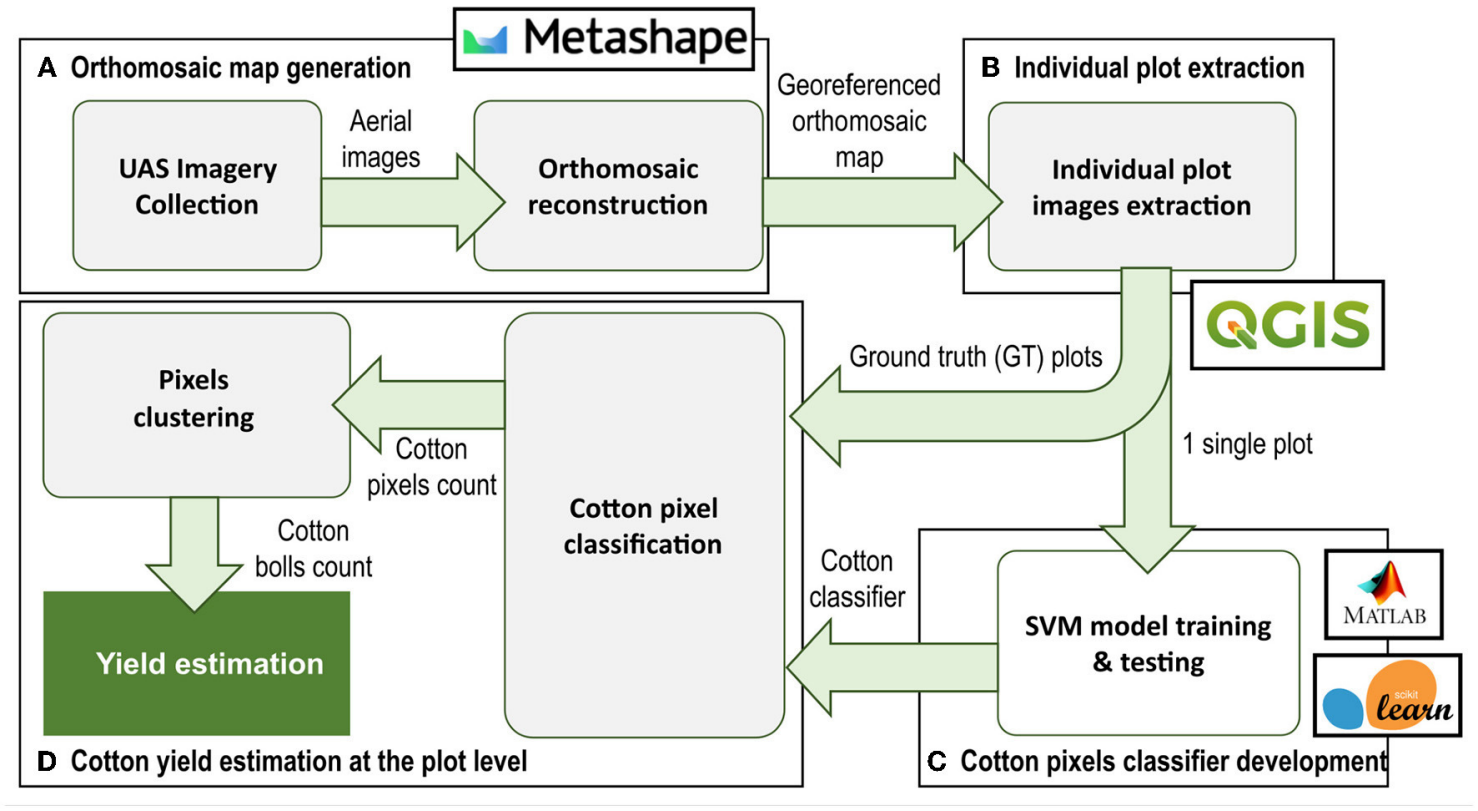
Data processing pipeline. The process pipeline includes 4 main steps: **(A)** Orthomosaic map generation, **(B)** Individual plot extraction, **(C)** Image pixel classifier development, and **(D)** Cotton yield estimation at plot level. Green arrows indicate the flow of data/information between processes.

The data processing pipeline for cotton yield estimation presented in this article (Figure 4) involved four main steps: (A) generation of an orthomosaic map of the entire field from the aerial images collected, (B) individual plot images extraction and pre-processing using image processing techniques, (C) development of an image pixel classifier based on SVM for cotton pixels segmentation at the plot level, and (D) cotton boll number estimation for each individual plot.

#### 2.3.1. Orthomosaic Map Generation and Individual Plot Image Extraction

An orthomosaic map was created from the RGB images using Agisoft Metashape software. A generic pair preselection with high accuracy setting was selected for photo alignment on Metashape software. By using the “*detect markers”* tool, all GCP markers were identified to georeference the images.

After applying a mild depth filtering and enabling interpolation, a digital elevation model (DEM) was generated using the dense point cloud from the estimated camera positions. Finally, using the DEM and mosaic as the blending mode, the orthomosaic map was obtained. To extract plot images from the orthomosaic map we used the open-source geographic information system (GIS) software Quantum GIS, version 3.8.2-Zanzibar (Open Source Geospatial Foundation, Beaverton, OR, U.S.). A vector layer with the boundaries of each plot was created manually and then clipped to the orthomosaic map to obtain an individual GeoTIF image file for each plot.

#### 2.3.2. Individual Plot Image Preprocessing

To reduce computation time and speed up the processing of individual plot images, preprocessing was implemented. Each individual plot image contains around 500,000 pixels. However, just a small portion of these pixels are meaningful for yield estimation, i.e., cotton-related pixels. As a general example, an image of a typical cotton crop plot will include plant leaves, weeds, and other plant matter (green to yellow color pixels); soil and other mineral matter with near-neutral hues (gray, brown, and tan color pixels); branches and other woody elements (light brown color pixels); and cotton pixels (shades of white color pixels). Hence, using traditional image processing techniques, all vegetation and soil-related pixels from the images could be potentially removed prior to pixel classification. A modified version of the Excess Green minus Excess Red Index (*ExG-ExR*) (Meyer and Neto, 2008) provided good results for removing vegetation pixels. To compute the modified index *ExGR*_*mod*_ index for the cotton plots, the following equation was used:


(1) $$\begin{array}{r} ExGR_{mod}=2.5\;\times \;G_{norm}-3\;\times \;R_{norm}-B_{norm} \end{array}$$

where *R*_*norm*_, *G*_*norm*_, and *B*_*norm*_ are the normalized *R, G*, and *B* color channel values respectively that were computed as in Meyer and Neto (2008).

Similarly, a new index that we called *Soil*_*Idx*_ based on the CIELAB color space was found useful to remove soil pixels without having any visible impact on the pixels associated to the cotton bolls. To compute this index the following equation was used:


(2) $$\begin{array}{r} Soil_{Idx}=0.5\;\times \;L_{norm}-2\;\times \;a_{norm}+b_{norm} \end{array}$$

where *L*_*norm*_, *a*_*norm*_, and *b*_*norm*_ are respectively the normalized L*, a*, and b* components of the CIELAB color space.

#### 2.3.3. SVM Classification Model Development

Cotton color is significantly different from most other elements in the field. Hence, pixel color can be used intuitively as a descriptor for cotton pixel segmentation. However, after a preliminary analysis of the RGB color component values of the image pixels, we found that some branches and woody elements in the background were almost indistinguishable from the cotton boll pixels, mainly because of shades and other light-blocking effects. This suggests that RGB color space information alone was not a robust enough descriptor to properly extract the cotton pixels, as previous studies have noted. Other color spaces, in particular HSV and CIELAB color models, increase invariance with respect to luminosity and lighting changes and are more robust than the RGB color space in relation to the presence of shadows (Hdioud et al., 2018). In this study, we applied an SVM model to classify image pixels using RGB, HSV, and CIELAB color spaces information. This information was used as feature descriptors to discriminate between cotton boll pixels and the rest of background pixels.

The SVM model was developed using the Scikit-learn library (Pedregosa et al., 2011) on the Jupyter Notebook interactive computing platform, version 6.1.4. To reduce the annotation burden, only a single plot image was used to extract features and create the dataset for model training. Initially, an 11-dimensional feature vector was extracted from each pixel. These vectors contained the location of the point in the image (row, col); and the values of the RGB, HSV and CIELAB color space components of the point (R, G, B, H, S, V, L*, a*, b*) for each pixel in the image. The Matlab Image Labeler app (The Math Works Inc., Natick, MA, U.S.) was used to annotate the image. This annotation tool enables the user to interactively draw pixel ROIs to label the boundaries of the visible cotton bolls to classify every image pixel into one of two target classes: cotton and non-cotton. The class of each pixel—1 for cotton pixels, and 0 for non-cotton pixels—was added to the features vector as the target column. To minimize the complexity of the model, a dimension reduction step was introduced to identify the best set of features. A recursive feature elimination (RFE) algorithm was applied for best features selection. The resulting dataset was divided into training and validation subsets with a ratio of 4:1. For model training, a radial basis function (RBF) kernel was used, the hyperparameter C was configured to be 1.0, and the hyperparameter γ was selected as “scale”.

To evaluate the performance of the SVM model for cotton pixels classification, accuracy, precision, recall, and Type I and Type II error rates were calculated. In addition, to provide a more comparable metric with other similar studies, the F1 score metric was also computed. The accuracy can be defined as the percentage of correct predicted pixels for the total number of pixels analyzed and can be calculated as follows:


(3) $$\begin{array}{r} Accuracy=\frac{TP+TN}{TP+TN+FP+FN}\times 100 \end{array}$$

where TP and TN—true positives and true negatives, respectively—are the number of pixels correctly classified for each class, and FP and FN—false positives and false negatives, respectively—are the number of misclassified pixels.

The precision metric measured the proportion of pixels classified as cotton pixels that were classified correctly. This metric accounts for the ability of the classifier to not label a pixel that is not cotton as cotton. In contrast, the Type I error rate indicates the probability of misclassifying an non-cotton object as cotton using the classifier. The precision and the Type I error rate can be calculated as follows:


(4) $$\begin{array}{r} precision=\frac{TP}{TP+FP}\times 100 \end{array}$$

(5) $$\begin{array}{r} Type\;I\;error\;rate=\frac{FP}{TP+FP}\times 100 \end{array}$$

The recall measured the proportion of cotton pixels that were classified correctly by the SVM model among all the actual cotton pixels in the image. This metric describes the ability of the classifier to find all cotton pixels. The Type II rate, in contrast, indicates the probability of misclassifying a cotton pixel as a non-cotton pixel. These metrics can be calculated as follows:


(6) $$\begin{array}{r} recall=\frac{TP}{TP+TN}\times 100 \end{array}$$

(7) $$\begin{array}{r} Type\;II\;error\;rate=\frac{TN}{TP+FP}\times 100 \end{array}$$

Finally, F1 score, as a function of precision and recall, conveyed the balance between those two metrics by taking their weighted average. It can be calculated using the following equation:


(8) $$\begin{array}{c} F1-Score=2\times \frac{precision\times recall}{precision+recall}\times 100 \end{array}$$

All these metrics ranged from 0% to 100%, 100% being related to the best performance.

#### 2.3.4. Yield Prediction Model Development and Evaluation Metrics

The developed SVM classifier was used to classify the cotton pixels presented on the unseen 45 plot images corresponding to the ground truth plots. After each image pixel was classified as a cotton or non-cotton point, a morphological erosion operation using a 3 × 3 elliptic structuring element, followed by a morphological dilation operation with a 5 × 5 rectangular structuring element were applied to eliminate noisy points and reduce the effect of pixels misclassification. Then, connected components labeling was applied to the binary image to count number of bolls. The connected components were computed using an 8-way pixel connectivity, where pixels are considered connected if they share any of the pixels that compose their respective Moore Neighborhood. These post-processing operations were performed using the OpenCV library (Bradski, 2000), version 4.5.3.

To evaluate the performance of the cotton yield prediction model, a linear regression analysis was performed between the estimated cotton boll numbers and the ground truth values. The coefficient of determination (*R*^2^) was used to check how closely the estimations mirrored the actual boll number at the individual plot level. Additionally, to facilitate performance comparison with other yield prediction studies that may use different scales, the normalized root mean squared error (NRMSE) was computed over the range of observed values—maximum cotton boll number minus minimum cotton boll number for the ground truth plots. The residuals were also computed to observe the difference between the ground truth data and the predicted values. Furthermore, to validate the performance of the yield prediction algorithm, the mean absolute percentage error (MAPE) was computed between the predicted number of cotton bolls and the ground truth measurements. These performance indices were computed using the following equations:


(9) $$\begin{array}{c} NRMSE=\frac{\sqrt{\frac{1}{N}\;\times \;\sum_{i=1}^{N}{\left(y_{i}-{\hat{y}}_{i}\right)}^{2}}}{y_{max}-y_{min}} \end{array}$$

(10) $$\begin{array}{r} MAPE\left(\%\right)=\frac{1}{N}\;\times \;\overset{N}{\underset{i=1}{\sum }}|\frac{y_{i}-{ŷ}_{i}}{y_{i}}|\;\times \;100 \end{array}$$

where *N* is the total number of data points used for the linear regression analysis (*N* = 45), *y*_i_ is the actual number of cotton bolls for the *i*^*th*^ ground truth plot, ŷ_i_ is the number of cotton bolls predicted by the SVM model for the image plot associated to the *i*^*th*^ ground truth plot, and $\bar{y}$ is the average number of cotton bolls per plot calculated from the ground truth values of all the 45 ground truth plots.

### 2.4. Genotype Analysis

The average number of predicted cotton bolls and the standard error (SE) for each genotype and population were calculated to evaluate the statistical accuracy of the yield estimations. To evaluate the effectiveness of the yield estimations, the null hypothesis of equal mean value of yield across all the commercial cultivars and breeding lines was tested using the one-way analysis of variance (ANOVA) at the significance level of 0.05. The statistical computing and graphics software R (R Core Team, 2020), version 4.0.3, was used for this test. After testing the effects due to genotype and its significance, the Fisher's Least Significant Difference (LSD) test was used to judge the likelihood that the observed differences between genotypes and populations comprised non-zero differences in yield performance. The LSD test was performed using the R package *agricolae* (de Mendiburu and Yaseen, 2020), version 1.3-5, to test differences among means of yield for all the genotypes.

## 3. Results

### 3.1. Individual Plot Image Extraction and Preprocessing

**Figure 5:**
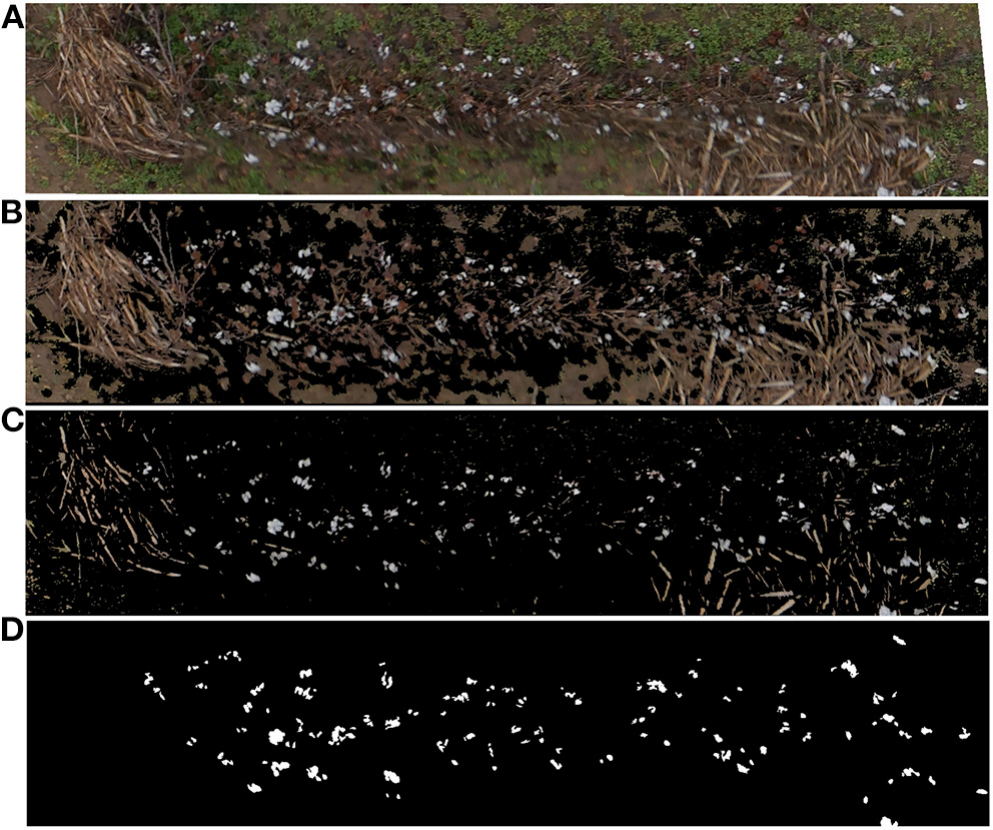
Individual plot image preprocessing results, **(A)** Raw RGB image extracted from the orthomosaic map, **(B)** Preprocessing results for the same plot image after removing vegetation pixels using the *ExGR*_*mod*_ index (Equation 1), **(C)** Preprocessing results for the same plot image after removing also bare soil pixels using the *Soil*_*Idx*_ index (Equation 2), **(D)** Binary mask used for SVM pixel classifier training. White color identifies cotton pixels. Black color identifies non-cotton pixels.

A total of 408 valid aerial images were used to generate the orthomosaic map (Figure 3). The 45 plots with the associated ground truth data were extracted manually from the orthomosaic map and saved as individual GeoTIF files. Figure 5 shows one of the extracted plot images. Specifically, this image was used for training the SVM classifier. The raw RGB image (Figure 5A) had 523,092 pixels. After removing the vegetation pixels using the *EXGR*_*mod*_ index (Equation 1), the new processed image (Figure 5B) had 231,447 pixels, which means that the total number of points to analyze was reduced to 44.25%. Finally, after removing the bare soil pixels using our *Soil*_*Idx*_ index (Equation 2), the processed image (Figure 5C) was down to 34,212 pixels, 6.54% of the raw image.

### 3.2. SVM Classification Model Development

A single image plot was used to train the SVM model. The image selected for developing the classifier included not only the cotton plants and the cotton bolls, but also other objects typically found in the crop such as old branches and other woody objects from previous crops, weeds, and soil (Figure 5A). The result of the annotation process was a binarized image mask in TIF file format (Figure 5D).

**Figure 6:**
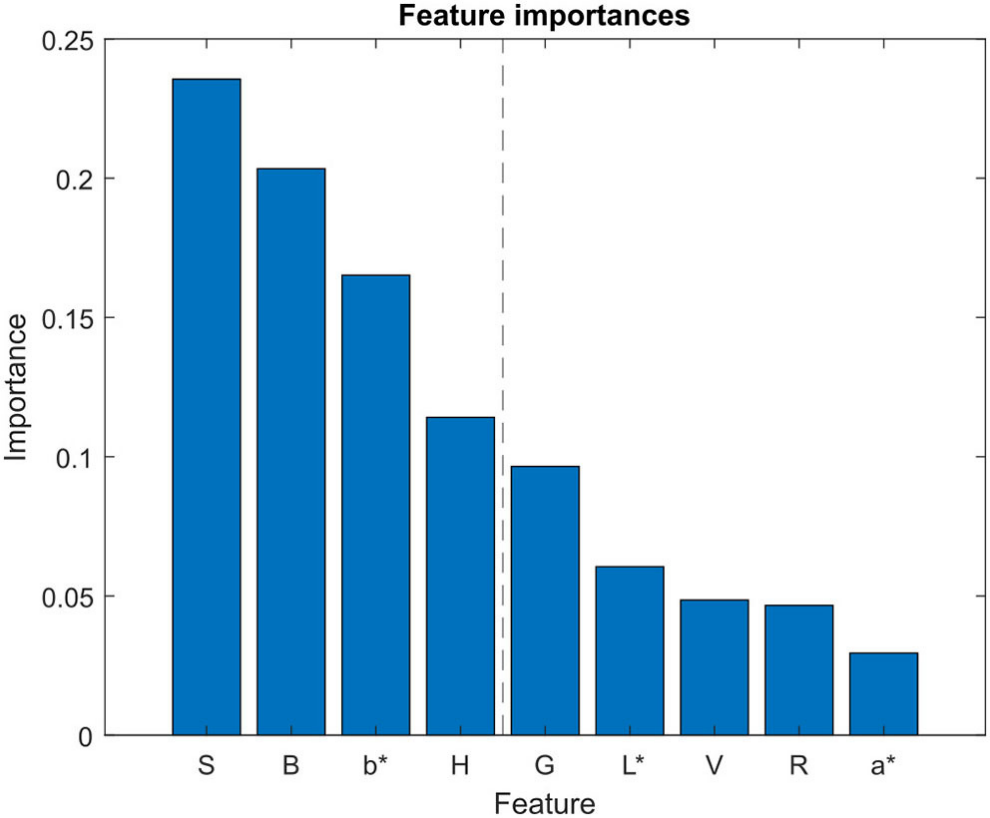
Feature selection analysis. Feature importances obtained using random forest algorithm for feature selection. Blue bars represent the importance of each feature in the classifier model. L*, a*, and b* refer to the CIELAB color space components.

For feature selection, just the 9 color channels (R, G, B, H, S, V, L*, a*, b*) were analyzed. The RFE algorithm was configured to select the 4 best features, by removing one feature at each iteration using a Random Forest (RF) classifier as the estimator. Results from the RF classifier (Figure 6) showed that the 4 most important features were S, B, b*, and H color components. These features were then used to create the training dataset, implying a reduction from the original 11-dimensional feature vector to a 4-dimensional vector. The resulting dataset, which contained 34,212 pixels of the preprocessed training image, was then split into the training subset (27,369 pixels) and the testing subset (6,843 pixels). By reducing the number of features from 11 to 4, the average time needed to classify the pixels of the training image plot was reduced a 13.3%, from 8.42 to 7.3 s.

**Figure 7:**
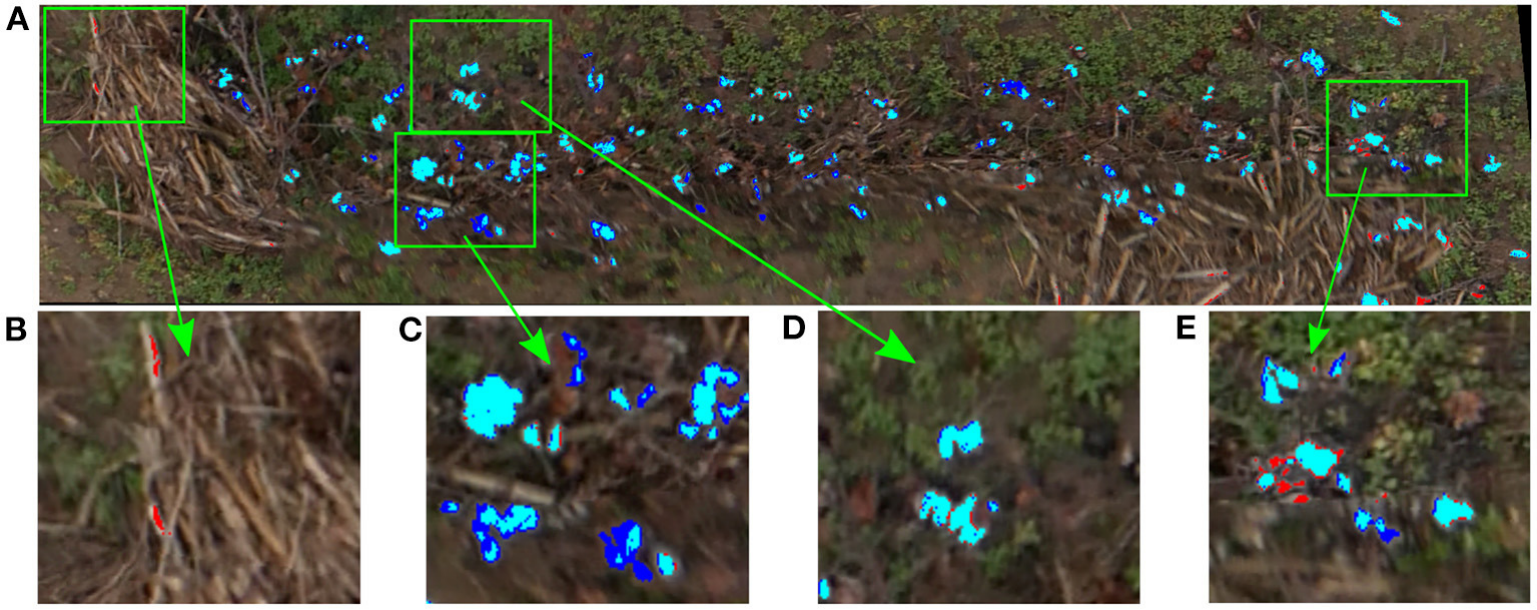
Inference results of the SVM classifier on the training plot image. Blue pixels were missed by the classifier (Type II errors); red pixels were misclassified as cotton by the classifier (Type I errors); cyan were correctly classified cotton pixels, **(A)** Training plot image classification results, **(B)** Non-cotton pixels misclassified as cotton (Type I errors), **(C)** Cotton boll pixels not fully detected (Type II errors), **(D)** Cotton boll pixels fully correctly detected, **(E)** Type I and Type II errors mixed together.

To analyze qualitatively the results of our cotton pixel classifier, a color code was used to identify image pixels. Figure 7 shows the inference results of the cotton classifier on the training plot image compared to the annotated mask. As can be seen, most of the cotton pixels are marked with cyan color, which indicates that they were correctly classified by the SVM model, i.e., true positives (Figures 7B–D). However, the classifier missed some of the cotton pixels presented in the image. A small portion of real cotton pixels were wrongly classified as non-cotton pixels, comprising false negatives or Type II errors and can be seen as pure blue pixels in Figures 7B,D. A smaller portion of non-cotton pixels were misclassified as cotton, comprising false positives or Type I errors and can be identified as pure red pixels in Figures 7A,D.

**Figure 8:**
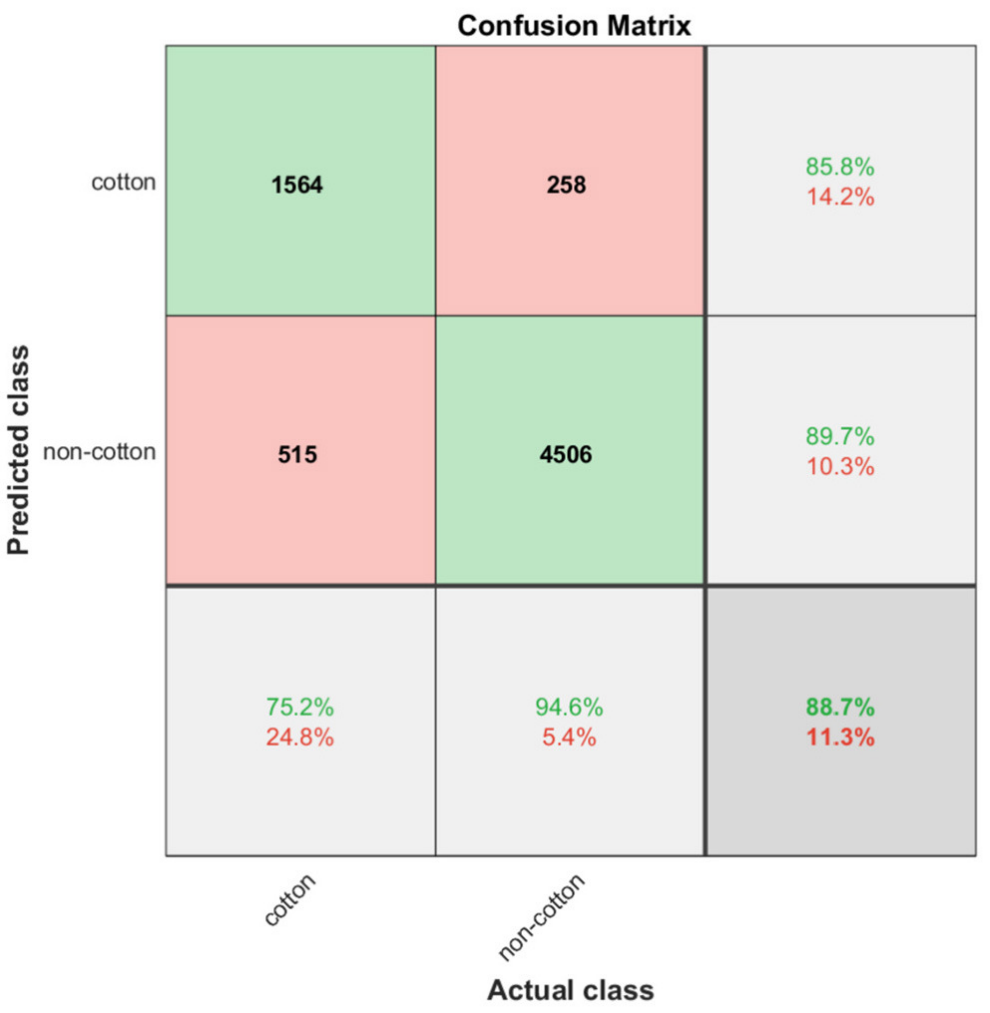
Confusion matrix chart. Green cells show the number of correct classifications by the SVM model. Red cells show the number of classification errors. Gray cells show overall performance of the model.

Quantitatively, the classification results of the trained SVM model on the testing subset are summarized in Figure 8. The column and row shown in gray indicate the classifier's overall performance. The cell in the bottom right of the plot shows the overall accuracy—correct predictions—of the pixel classifier. The model achieved an accuracy of 88.7%; 22.9% of the 6843 testing pixels were correctly classified as cotton and 65.8% of all testing pixels were correctly classified as non-cotton. Only 11.3% of predictions were wrong; 7.5% of the cotton pixels were incorrectly classified as non-cotton and 3.8% of non-cotton pixels were incorrectly classified as cotton. The column on the far right of the plot shows the percentages of all pixels predicted to belong to each class that were correctly and incorrectly classified. Accounting only for the positive class identification, the upper right cell indicates the precision and the rate of Type I errors of our model. With 1564 out of 1821 cotton pixels being correctly predicted, the SVM classifier achieved a precision of 85.8%, and a Type I error rate of 14.2%. The row at the bottom of the plot shows the percentages of all the pixels that belonging to each class were correctly and incorrectly classified. The bottom left cell indicates the recall and the rate of Type II errors of our classifier. Out of 2079 actual cotton pixels, the model achieved a recall of 75.2% and a Type II error rate of 24.8%. Finally, the image pixel classifier achieved an F1 score of 80.2% at detecting cotton pixels on the testing subset.

### 3.3. Plot-Level Cotton Yield Estimation Model Development

**Figure 9:**
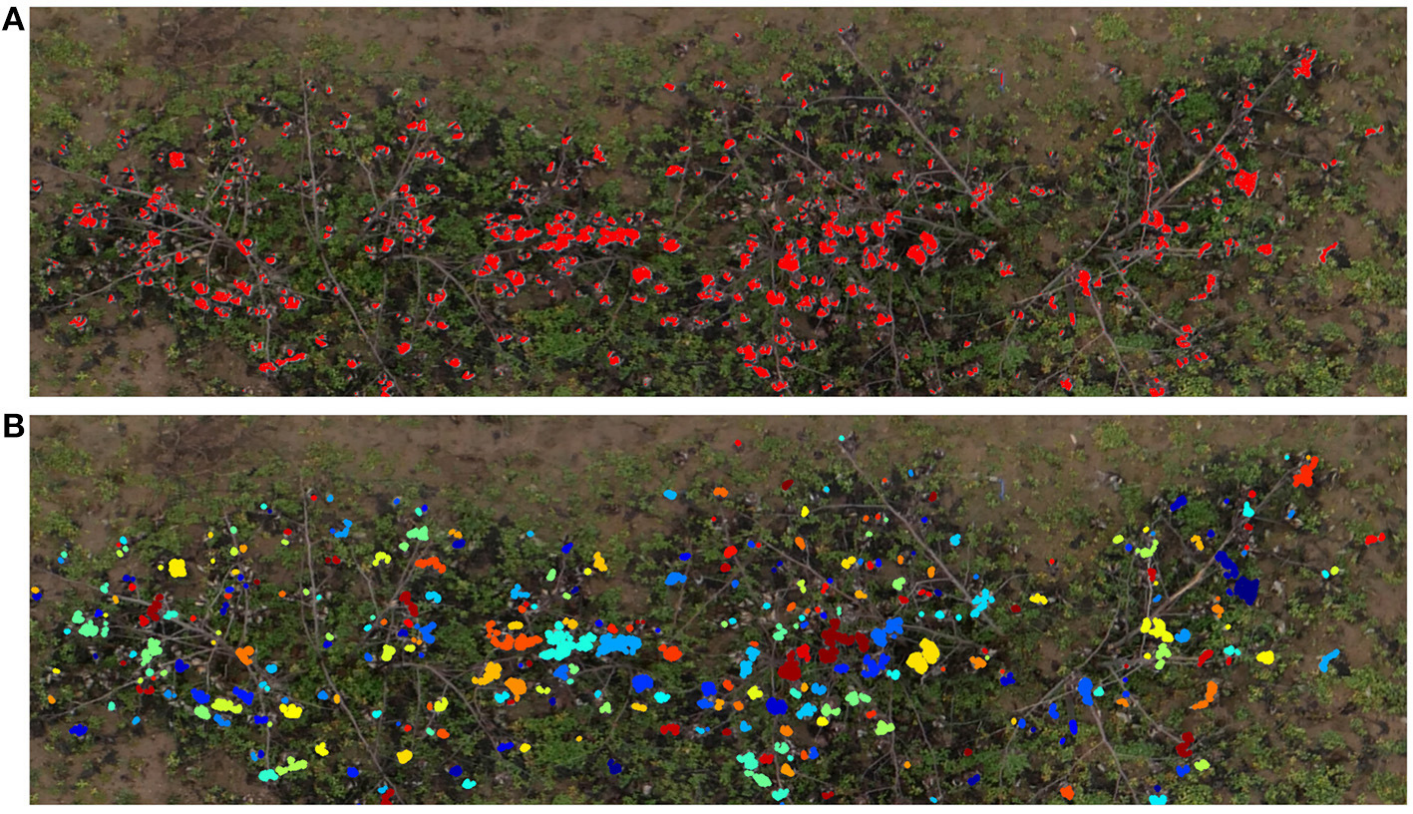
Cotton boll inference results. Cotton pixels segmentation and clustering results for a representative plot (Plot ID 45003), **(A)** SVM classifier inference results. Red pixels represent the cotton pixels detected by the SVM classifier as cotton, **(B)** Cotton boll clustering results. Each cluster is identified by a unique color.

The pixel classifier we developed was then used to extract the cotton pixels from the 45 individual images associated with the digital ground truth plots. It was able to detect subtle color changes, and was robust enough to avoid misclassifying most of the woody elements and the soil (Figure 9A). After applying the image post-processing steps and the clustering algorithm, we obtained an estimation of the number of cotton bolls for each image. Figure 9B shows the clustering results of a sample image plot extracted from the orthomosaic map. A total of 344 different cotton bolls (pixel clusters) were identified on this particular image plot. Each one of these clusters are identified by a unique color to facilitate the visual analysis.

**Figure 10:**
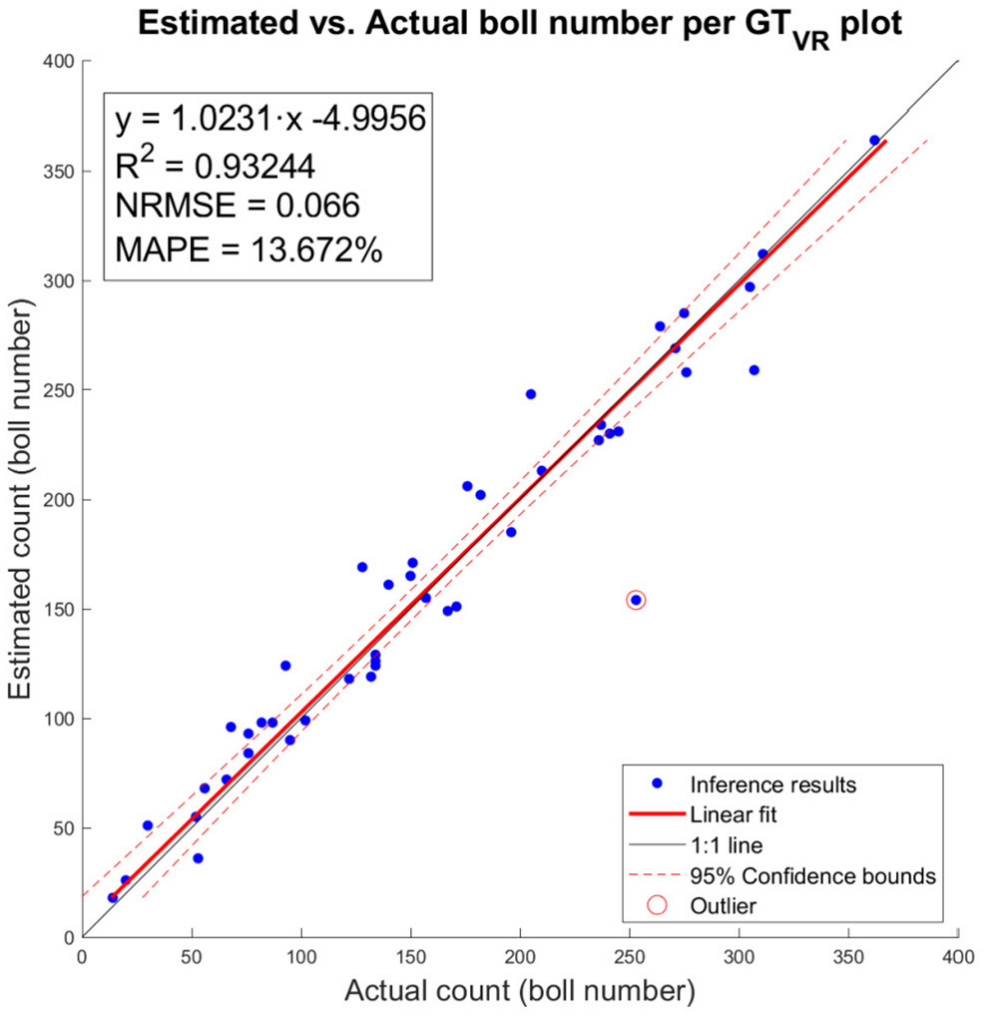
Estimated vs. actual number of cotton bolls per ground truth plot (GT_VR_). Blue dots represent inference results of our model. Red line represents the linear fit. Red dotted lines represent the 95% confidence interval for the fit. One outlier is marked with a circle surrounding the data point.

The number of cotton bolls estimated for the 45 individual image plots analyzed was regressed against its ground truth measurement (Figure 10). The estimations of number of cotton bolls at the plot level shows a strong linear relationship (*R*^2^ = 0.932) with the ground truth measurements. This trend is consistent at different numbers of cotton bolls, which indicates that our pixel classifier and clustering algorithm adapted to the changing scenes and was able to segment properly the cotton bolls from both low yielding plots and high yielding plots. The analysis of residuals showed randomly dispersed points around the horizontal axis with no apparent pattern, which indicates that the linear model was a good fit for the input data. Only one data point does not follow the regular distribution (red circled point on Figure 10) and given its value is more than three standard deviations from the mean, it was identified as an outlier. Our model achieved a MAPE of 13.672% at detecting cotton bolls and a normalized RMSE value of 0.066 over the range of observed cotton bolls.

### 3.4. Web API Deployment

**Figure 11:**
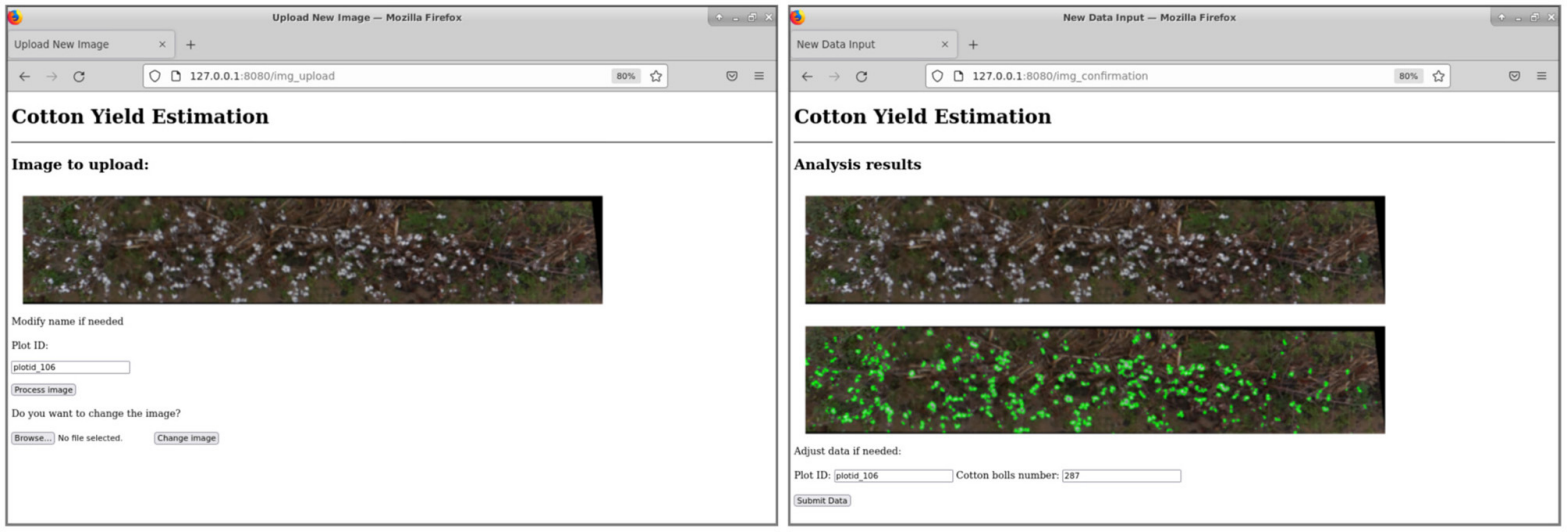
Web API for automatic image analysis.

A web-based API (web app) was developed to integrate the developed pipeline into a more usable interface with the aim of improving automation and usability (Figure 11). The web app consisted of three basic functions to process each input plot image: the preprocessing steps (vegetation and soil pixels removal), the SVM classifier deployment (creation of features and SVM pixel classification), and the final cotton boll number estimation (morphological image processing operations and connected components labeling algorithm). The SVM classifier and the clustering algorithm were deployed using Flask as the core of the API, in a dockerized environment. A Docker container image of the web app is available on Docker Hub in the repository https://hub.docker.com/r/javirodsan/yieldestimation. Additionally, we will provide the code and some sample images for testing at https://github.com/Javi-RS/Cotton_Yield_Estimation.

### 3.5. Genotype Analysis Results

The mean number of predicted cotton bolls and the SE for each genotype are summarized in Table 2. Results show that the cotton yield estimations produced by the proposed method has relatively low SEs for each cultivar and breeding line, which indicates that the means of the yield for the different genotypes are centered around the population mean, and hence, the sampled plots are representative of the population.

**Table 2:** Statistical analysis summary of predicted yield for commercial cultivars and breeding lines.

**Genotype**	**Sample size**	**Mean (boll number)**	**SE**	**Groups**
UA48	10	237.300	26.755	a
Acala1517-98	10	211.500	25.937	ab
GA230	10	199.100	21.969	abc
DeltaPine393	10	198.300	23.137	abc
L^†^	16	179.625	15.264	abcd
N^†^	20	178.800	32.627	bcd
FM832	10	176.800	19.960	bcd
Q^†^	40	172.525	11.224	bcd
S^†^	184	166.576	5.380	bcd
O^†^	22	163.591	18.585	bcd
M^†^	8	158.875	32.627	bcd
K^†^	44	158.364	10.682	cd
TAM94L25	10	156.300	18.049	cd
R^†^	44	151.614	10.072	cd
J^†^	18	143.889	14.703	cd
P^†^	32	137.594	11.275	d

*Data are sorted from higher to lower yield by genotype. Genotypes are grouped according to the probability of means differences and alpha level (0.05). Cultivars and breeding lines with the same letter are not significantly different*.

† *Indicates breeding line populations, comprised of samples of progeny from crosses between different mutant lines described in Patel et al. (2014)*.

The ANOVA test identified significant differences between the means of estimated yield for cotton genotypes, with *F*_(15, 472)_ = 1.874 and *p* < 0.05. Thus, the null hypothesis of equal mean value of yield across all the genotypes can be rejected, which suggests that our methodology was effective in identifying differences in yield. The Fisher's LSD identified statistical differences between the estimation of average yield for the cultivars, with UA48 and Acala1517-98 being significantly higher than TAM94L25, while FM832 being significantly lower than UA48. Regarding the mutant-derived populations, L and N populations had the highest yielding, and P had the lowest yielding, with other groups in-between. While the LSD test was able to identify significant differences of means of yield among the commercial cultivars, individual breeding lines had only two replications which provided insufficient evidence for definitive ranking.

## 4. Discussion

Estimating cotton yield before harvesting would assist breeders to identify highly productive genotypes without incurring the time and cost of actually harvesting the field. Our study demonstrated that the number of cotton bolls present on each individual plot of a field can be estimated accurately by using RGB images captured from a drone flight at a low altitude. This approach can be used to quickly estimate yield at the plot level and would allow cotton breeders analyze large variety trials efficiently, especially with higher levels of replication as were used for the cultivars. However, additional data would be needed to confirm its usability on experimental breeding lines.

### 4.1. Comparison With Other Studies

As opposed to previous methods, our method used a supervised machine learning model to classify the pixels in the image instead of using traditional global thresholding techniques. Approaches for cotton yield estimation based on traditional image processing techniques (Huang et al., 2016; Yeom et al., 2018; Dodge, 2019) usually assume that cotton has a distinctive spectral response that enables the easy discrimination of cotton bolls from the rest of the elements of the crop just by using a threshold value in one or more of the RGB channels. However, in a real-case scenario the illumination conditions can change considerably during data collection, and often the range of image intensities of the color channels for the cotton bolls are similar to other crop elements. Although some studies have applied adaptive threshold techniques or have included prior preprocessing steps to minimize the limitations of global thresholding techniques (Maja et al., 2016), these approaches are not flexible enough to cope with the variability of reflectance across a field, and hence their accuracy is limited. Our method uses an SVM model based on 4 image channels to segment cotton-related pixels. Machine learning techniques are more flexible than traditional image processing methods at finding patterns on data with non-obvious relationships. A recent study (Ashapure et al., 2020) has already investigated the use of machine learning techniques to estimate cotton yield. However, the focus of their study was to find the relationship between cotton yield and the parameters of the crop during the course of the season, not near harvest. Therefore, the potential of this approach for later growth stages might be limited because it includes crop features related to the canopy status. Our approach was developed to be applied after crop defoliation (which commonly precedes cotton harvest) to reduce the effects of occlusions by leaves and maximize cotton boll visibility. Although this limits the applicability of our method to the pre-harvesting time frame, this is the period during which breeders evaluate the overall performance of new breeding lines, so it can be considered one of the key stages in the selection trials.

### 4.2. Type I and II Errors Analysis

**Figure 12:**
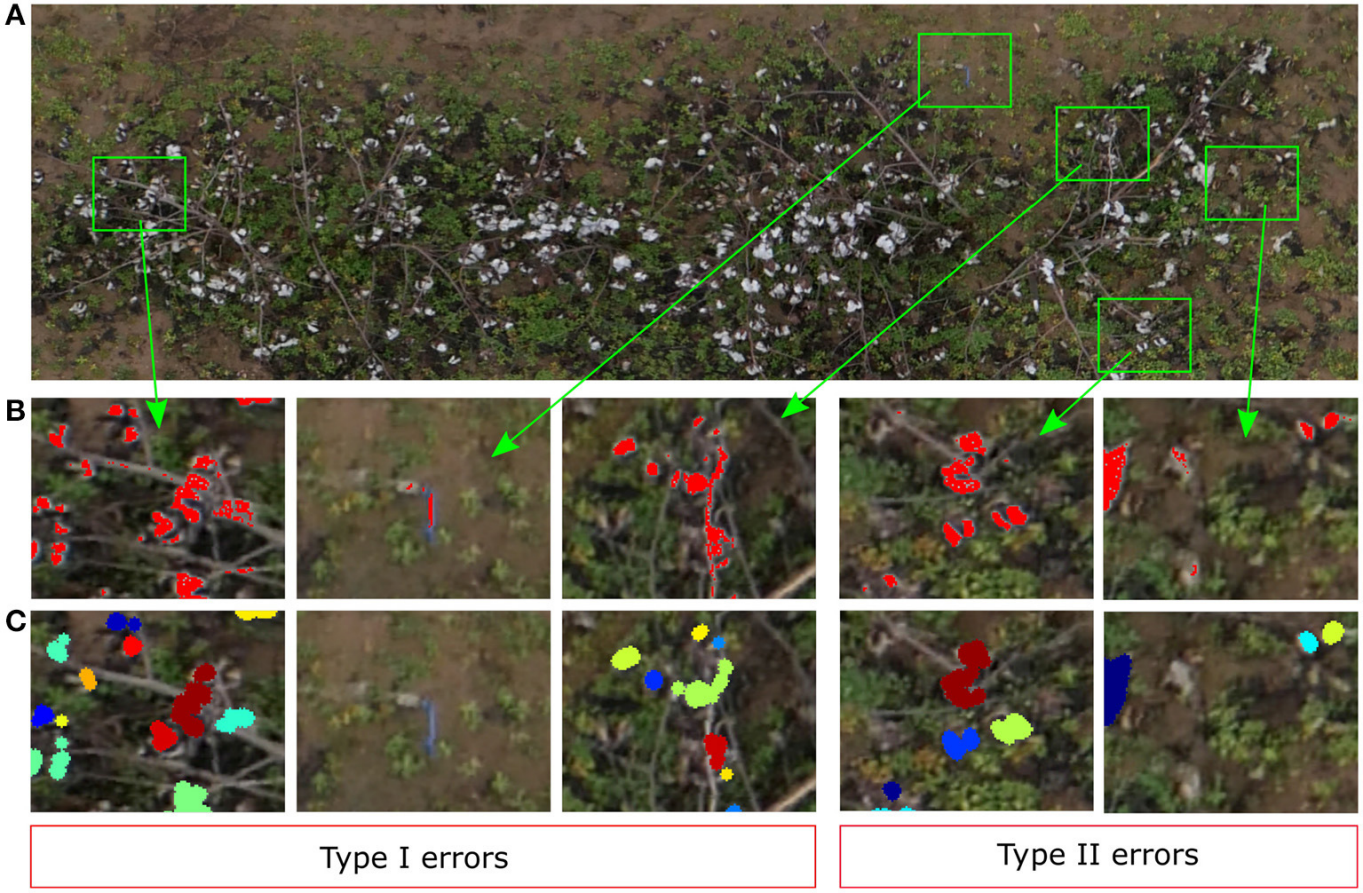
Qualitative analysis of the yield estimator. **(A)** Original image plot (Plot ID 45003), **(B)** Zoomed-in view to Type I and Type II errors at segmenting cotton pixels. Red represents the pixels segmented by the SVM classifier as cotton pixels, **(C)** Zoomed-in view to the same zones as **(B)** to visualize the clustering results. Different colors identify different clusters (cotton bolls).

Our SVM classifier was able to segment the cotton pixels accurately from the input images, showing a promising overall performance for the training image (Figure 7). As we have commented in section 3.2, the classifier made some Type I errors (false positives), and Type II errors (false negatives). In our context we tried to minimize the Type I errors, i.e., the number of background pixels wrongly classified as cotton pixels. We aimed to detect all the cotton pixels in the image, but we did not want to overestimate them. Usually, the number of cotton boll pixels in a plot image is much lower than the number of pixels from other parts of the plants and background. Hence, the chances of misclassifying non-cotton pixels are higher. Figure 12 shows the performance of the SVM classifier model on an unseen plot image. Type I errors were mainly caused by elements of the scene with a spectral response similar to the cotton bolls. As shown in Figure 12B, some branches and other foreign objects in the field were highly reflective and they were misclassified as cotton pixels.

Type II errors were caused primarily by dark cotton pixels in the image that were not properly detected by the classifier. Bolls from the lower parts of the canopy were less reflective than those from the top parts with more light. Therefore, the image pixels were usually darker on these parts, and the SVM classifier was not able to detect completely all the cotton pixels of some cotton bolls (Figure 12B). These zones are usually small, and the image post-processing operations tend to remove them before clustering (Figure 12C). Even though there were some obvious classification errors, most of the cotton bolls detected by our algorithms were true cotton bolls. These errors do not necessarily have a substantial effect on our estimations of yield because it was expected that not all the cotton bolls can be seen from downward images.

The morphological operations enhanced the appearance of the cotton bolls in the binarized images and reduced the influence of the SVM Type I errors on the cotton boll estimation. The small elliptic structuring element used for the erosion operation contributed to removing isolate pixels and small pixel clusters associated to branches and other wrongly classified elements, and hence the pixel clustering step performed relatively well at isolating cotton bolls and correcting Type I errors (Figure 12C). A larger element for the dilation operation aided to extend the boundaries of the rest of cotton pixels to eliminate gaps between close pixels and consolidate cotton bolls (Figure 12C).

### 4.3. Limitations

We used a digital RGB camera to collect the aerial images, which internally processes the data from the image sensor and performs the JPEG compression to save the image in a removable storage media. These images are stored without a previous radiometric correction. We tried to minimize the effect of the illumination on the data collection day by using an automatic color balance compensation. Although the AWB compensation was adequate for our data analysis, it might not be a universal solution for all the possible illumination conditions in the field. Therefore, the use of our model directly to images collected at different times may be limited if the atmosphere and solar radiation greatly differ from those on our data collection day. However, since the method we proposed is relatively fast and easy to use, retraining the SVM model with new data from the specific collection day can be feasible.

**Figure 13:**
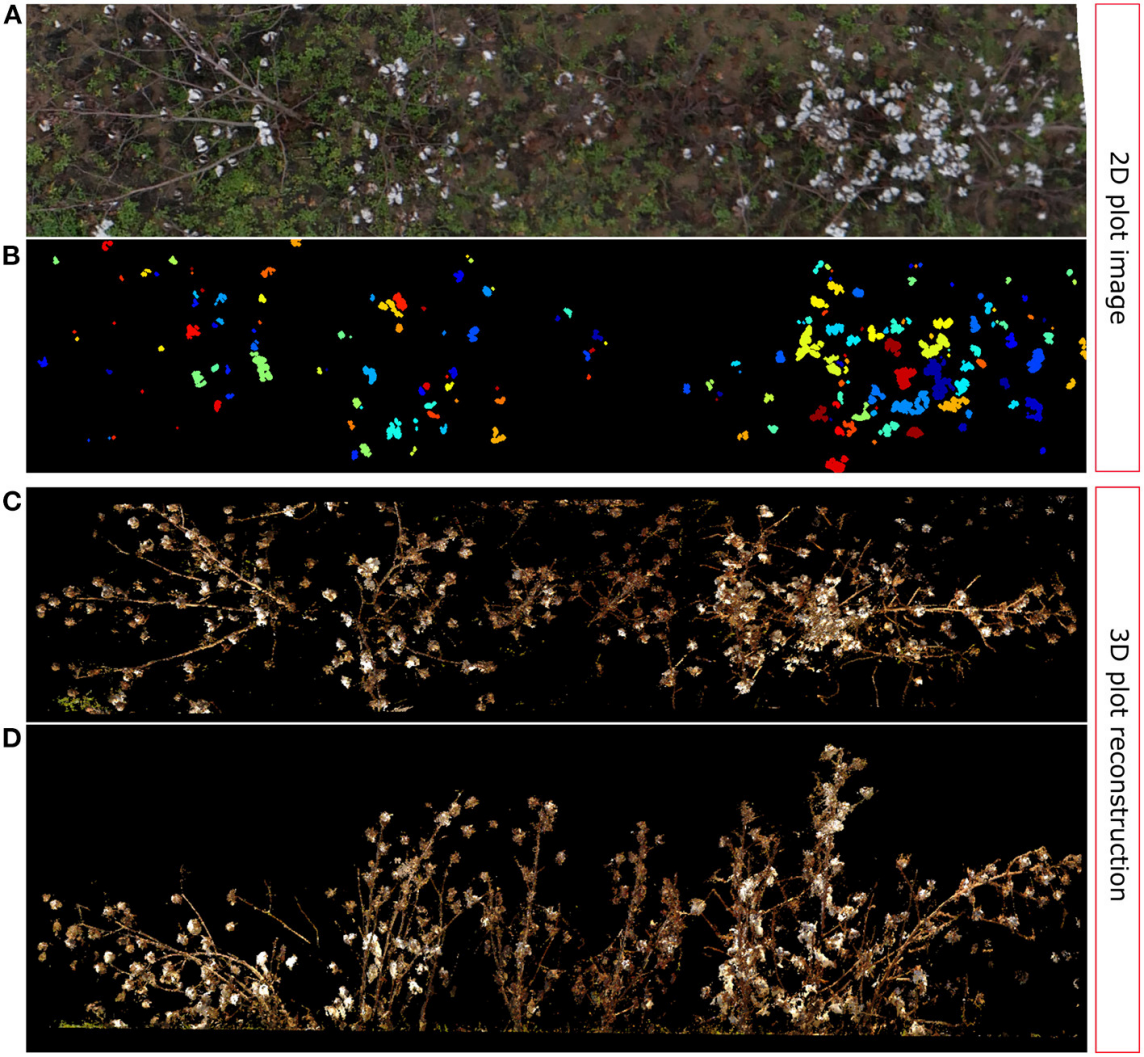
Outlier analysis: 2D vs. 3D comparison. **(A)** Original image plot (Plot ID 41028), **(B)** Cotton boll clustering results; each cluster is identified by a unique color, **(C)** TLS reconstructed 3D point cloud: Top view, **(D)** TLS reconstructed 3D point cloud: Frontal view.

Additionally, as we noted in section 3.3, one of the points in the data set was identified as an outlier during the linear regression analysis (red circled point in Figure 10). By further analyzing this particular data point and the associated plot (Figure 13), we can determine that these kind of errors are caused by one of the main limitations of 2D image analysis: lose of depth information. The SVM classifier detected the cotton pixels in the orthomosaic image fairly well. Moreover, the clustering algorithm was able to find and segment properly some of the cotton bolls (Figure 13B). However, the high density of cotton bolls in this plot made the cotton pixels to appear close together on the 2D aerial image (Figure 13A), which prevented our clustering algorithm from segmenting all the cotton bolls properly. Therefore, the estimation of number of cotton bolls for this plot was not accurate—only 154 out of 253 cotton bolls were detected. If we compare this image with its 3D point cloud counterpart, we can see that a substantial number of the cotton bolls were located in almost vertical branches (Figure 13D), which made the lower cotton bolls to be heavily occluded by the rest when looking from the top (Figure 13C). In a 2D image all the pixels are contained in the same plane. This lack of depth information impeded our algorithm to identify cotton pixels at different height levels and led to underestimating the number of cotton bolls.

### 4.4. Future Work

Most of the image processing can be performed automatically without any supervision. However, the extraction of plot images from the orthomosaic map was carried out manually. A future research direction could investigate the feasibility of automatically separating the plots from the orthomosaic map using only geographic information. This would improve the efficiency of this methodology and would contribute to improving the throughput for breeding purposes. Additional research could also be performed to improve cotton pixel classification, including the use of more advanced deep learning methods such convolutional neural networks (CNN) that could discriminate cotton pixels from the rest of elements by automatically integrating spatial and morphological information as additional features without needing to design them manually. Even though the methodology presented here was developed and tested using individual plot images, it could be modified easily to perform yield estimations on production fields because of its ease of use. Some minor modifications on the processing pipeline would make it suitable for production fields. Instead of extracting entire plots, the orthomosaic map could be divided into several sections or cells using grids with fixed dimensions. Then, our method could be used to estimate the yield from each cell and then aggregate the estimations to have an estimation of the total production of the entire field. However, further studies will need to be carried out to validate this approach on different crop densities. Additionally, our model was able to detect cotton bolls consistently from almost all the plots of the field. However, we didn't correct the images atmospherically, which may affect the direct application of our model to images collected under different conditions. We will investigate how to further improve our pipeline by including a preprocessing step to correct radiometrically the images and make our method agnostic to illumination lighting conditions and imaging sensors. Finally, we were able to use digital ground truth techniques based on 3D information to demonstrate some of the limitations of 2D approaches for estimating yield from aerial imagery. We will investigate the feasibility of using 3D crop analysis to overcome these limitations and its viability to estimate other crop traits.

## 5. Conclusions

This study presents a cost-effective approach for estimating cotton yield production from images collected using a drone and a conventional RGB camera. A supervised machine learning classifier based on an SVM model was trained using only a single plot image. Since this approach requires the annotation of only one RGB image, it reduces the complexity and time needed for model deployment. The classifier demonstrated to be robust to changing scenes and discriminated accurately the cotton pixels in individual plot images with different number of cotton bolls. Consequently, reliable cotton boll counting was obtained. In addition, the methodology was found to be effective in identifying the differences in yield among different commercial cultivars and breeding lines. Overall, the proposed method can help improve the efficiency of decision making for breeding programs and optimize the use of resources by speeding up the analysis of entire field trials. Future work will focus on automating the extraction of plot images, as well as on the application of 3D-based approaches and more advanced machine learning methods to improve cotton boll detection.

## Data Availability Statement

The raw data supporting the conclusions of this article will be made available by the authors, without undue reservation.

## Author Contributions

JR-S contributed to the conceptualization of the idea, methodology, investigation, software development, validation, formal analysis, data curation, writing—original draft preparation, visualization, and project administration. CL contributed to the conceptualization, methodology, provision of resources, supervision, project administration, and funding acquisition. AP contributed to the methodology, provision of resources, and funding acquisition. All authors contributed to manuscript revision, read, and approved the submitted version.

## Funding

This work was funded by the National Science Foundation Growing Convergence Research (Award No. 1934481) and the Georgia Cotton Commission.

## Conflict of Interest

The authors declare that the research was conducted in the absence of any commercial or financial relationships that could be construed as a potential conflict of interest.

## Publisher's Note

All claims expressed in this article are solely those of the authors and do not necessarily represent those of their affiliated organizations, or those of the publisher, the editors and the reviewers. Any product that may be evaluated in this article, or claim that may be made by its manufacturer, is not guaranteed or endorsed by the publisher.

## References

[B1] Ashapure A. Jung J. Chang A. Oh S. Yeom J. Maeda M.. (2020). Developing a machine learning based cotton yield estimation framework using multi-temporal UAS data. ISPRS J. Photogrammetry Remote Sens. 169, 180–194. 10.1016/j.isprsjprs.2020.09.015

[B2] Barbedo J. G. A.. (2019). A review on the use of unmanned aerial vehicles and imaging sensors for monitoring and assessing plant stresses. Drones 3. 10.3390/drones3020040

[B3] Bourland F. Myers G. O. (2015). “Conventional cotton breeding,” in Cotton, eds D. D. Fang and R. G. Percy (Madison, WI: John Wiley & Sons, Ltd.), 205–228. 10.2134/agronmonogr57.2013.0025

[B4] Bowman D. Bourland F. Myers G. Wallace T. Caldwell D. (2004). Visual selection for yield in cotton breeding programs. J. Cotton Sci. 8, 62–68.

[B5] Bradski G.. (2000). The OpenCV Library. Dr. Dobb's Journal of Software Tools. Available online at: https://github.com/opencv/opencv/wiki/CiteOpenCV

[B6] Chu T. Chen R. Landivar J. Maeda M. Yang C. Starek M. (2016). Cotton growth modeling and assessment using unmanned aircraft system visual-band imagery. J. Appl. Remote Sens. 10, 1–17. 10.1117/1.JRS.10.036018

[B7] Cortes C. Vapnik V. (1995). Support vector networks. Mach. Learn. 20, 273–297. 10.1007/BF00994018

[B8] de Mendiburu F. Yaseen M. (2020). agricolae: Statistical Procedures for Agricultural Research. R package version 1.4.0. Available online at: https://cran.r-project.org/web/packages/agricolae/index.html

[B9] Dodge W.. (2019). Image based yield estimation in cotton using UAS (Ph.D. dissertation). Texas Tech University, Lubbock, TX, United States.

[B10] Drucker H. Burges C. J. C. Kaufman L. Smola A. J. Vapnik V. (1996). “Support vector regression machines,” in NIPS, eds M. Mozer, M. I. Jordan, and T. Petsche (Cambridge, MA: MIT Press), 155–161.

[B11] Feng A. Zhou J. Vories E. D. Sudduth K. Zhang M. (2020). Yield estimation in cotton using UAV-based multi-sensor imagery. Biosyst. Eng. 193, 101–114. 10.1016/j.biosystemseng.2020.02.014

[B12] Garcia-Ruiz F. Sankaran S. Maja J. M. Lee W. S. Rasmussen J. Ehsani R. (2013). Comparison of two aerial imaging platforms for identification of huanglongbing-infected citrus trees. Comput. Electron. Agric. 91, 106–115. 10.1016/j.compag.2012.12.002

[B13] Hdioud B. Tirari M. E. H. Thami R. O. H. Faizi R. (2018). Detecting and shadows in the HSV color space using dynamic thresholds. Bull. Electric. Eng. Inform. 7, 70–79. 10.11591/eei.v7i1.893

[B14] Huang Y. Howard J. B. Ruixiu S. Steven J. T. Tomonari F. M. (2016). Cotton yield estimation using very high-resolution digital images acquired with a low-cost small unmanned aerial vehicle. Trans. ASABE 59, 1563–1574. 10.13031/trans.59.11831

[B15] Huang Y. Sui R. Thomson S. Fisher D. K. (2013). Estimation of cotton yield with varied irrigation and nitrogen treatments using aerial multispectral imagery. International J. Agric. Biol. Eng. 6, 37–41. 10.3965/j.ijabe.20130602.005

[B16] Jung J. Maeda M. Chang A. Landivar J. Yeom J. McGinty J. (2018). Unmanned aerial system assisted framework for the selection of high yielding cotton genotypes. Comput. Electron. Agric. 152, 74–81. 10.1016/j.compag.2018.06.051

[B17] Liu S. Whitty M. A. (2015). Automatic grape bunch detection in vineyards with an svm classifier. J. Appl. Log. 13, 643–653. 10.1016/j.jal.2015.06.001

[B18] Maja J. M. J. Campbell T. Camargo Neto J. Astillo P. (2016). “Predicting cotton yield of small field plots in a cotton breeding program using UAV imagery data,” in Autonomous Air and Ground Sensing Systems for Agricultural Optimization and Phenotyping, eds J. Valasek and J. A. Thomasson (Baltimore, MD), 98660C. 10.1117/12.2228929

[B19] Meyer G. Neto J. C. (2008). Verification of color vegetation indices for automated crop imaging applications. Comput. Electron. Agric. 63, 282–293. 10.1016/j.compag.2008.03.009

[B20] Patel J. D. Wright R. J. Auld D. Chandnani R. Goff V. H. Ingles J.. (2014). Alleles conferring improved fiber quality from EMS mutagenesis of elite cotton genotypes. Theoret. Appl. Genet. 127, 821–830. 10.1007/s00122-013-2259-6 24374351

[B21] Pedregosa F. Varoquaux G. Gramfort A. Michel V. Thirion B. Grisel O.. (2011). Scikit-learn: machine learning in python. J. Mach. Learn. Res. 12, 2825–2830. 10.48550/arXiv.1201.0490

[B22] R Core Team (2020). R: A Language and Environment for Statistical Computing. R Foundation for Statistical Computing, Vienna.

[B23] Raza S.-A. Prince G. Clarkson J. P. Rajpoot N. M. (2015). Automatic detection of diseased tomato plants using thermal and stereo visible light images. PLoS ONE 10, e0123262. 10.1371/journal.pone.0123262 25861025 PMC4393321

[B24] Rumpf T. Mahlein A. K. Steiner U. Oerke E. C. Dehne H. W. Plümer L. (2010). Original paper: early detection and classification of plant diseases with support vector machines based on hyperspectral reflectance. Comput. Electron. Agric. 74, 91–99. 10.1016/j.compag.2010.06.009

[B25] Song Y. Glasbey C. A. Horgan G. W. Polder G. Dieleman J. A. van der Heijden G. (2014). Automatic fruit recognition and counting from multiple images. Biosyst. Eng. 118, 203–215. 10.1016/j.biosystemseng.2013.12.008

[B26] Townsend T. Sette J. (2016). “Natural fibres and the world economy,” in Natural Fibres: Advances in Science and Technology Towards Industrial Applications, eds R. Fangueiro and S. Rana (Dordrecht: Springer), 381–390. 10.1007/978-94-017-7515-1_30

[B27] Vapnik V. Golowich S. E. Smola A. J. (1997). “Support vector method for function approximation, regression estimation and signal processing,” in Advances in Neural Information Processing Systems 9 – *Proceedings of the 1996 Neural Information Processing Systems Conference (NIPS 1996)*, eds M. Mozer, M. I. Jordan, and T. Petsche (Dever, CO; Cambridge, MA: MIT Press), 281–287.

[B28] Xia L. Zhang R. Chen L. Huang Y. Xu G. Wen Y.. (2019). Monitor cotton budding using SVM and UAV images. Appl. Sci. 9. 10.3390/app9204312

[B29] Xu R. Li C. Paterson A. H. (2019). Multispectral imaging and unmanned aerial systems for cotton plant phenotyping. PLoS ONE 14, e0205083. 10.1371/journal.pone.0205083 30811435 PMC6392284

[B30] Yeom J. Jung J. Chang A. Maeda M. Landivar J. (2018). Automated open cotton boll detection for yield estimation using unmanned aircraft vehicle (UAV) data. Remote Sens. 10. 10.3390/rs10121895

